# Early onset of hypertension and serum electrolyte changes as potential predictive factors of activity in advanced HCC patients treated with sorafenib: results from a retrospective analysis of the HCC-AVR group

**DOI:** 10.18632/oncotarget.7444

**Published:** 2016-02-17

**Authors:** Andrea Casadei Gardini, Emanuela Scarpi, Giorgia Marisi, Francesco Giuseppe Foschi, Gabriele Donati, Emanuela Giampalma, Luca Faloppi, Mario Scartozzi, Nicola Silvestris, Marcello Bisulli, Jody Corbelli, Andrea Gardini, Giuliano La Barba, Luigi Veneroni, Stefano Tamberi, Stefano Cascinu, Giovanni Luca Frassineti

**Affiliations:** ^1^ Department of Medical Oncology, Istituto Scientifico Romagnolo per lo Studio e Cura dei Tumori (IRST) IRCCS, Meldola, Italy; ^2^ Unit of Biostatistics and Clinical Trials, IRST IRCCS, Meldola, Italy; ^3^ Biosciences Laboratory, IRST IRCCS, Meldola, Italy; ^4^ Internal Medicine, Hospital of Faenza, AUSL Romagna, Faenza, Italy; ^5^ Internal Medicine, Infermi Hospital, AUSL Romagna, Rimini, Italy; ^6^ Radiology Unit, Infermi Hospital, AUSL Romagna, Rimini, Italy; ^7^ Department of Medical Oncology, University Hospital of Ancona, Polytechnic University of Marche, Ancona, Italy; ^8^ Departments of Medical Oncology, University Hospital Cagliari, Cagliari, Italy; ^9^ Medical Oncology Unit, Cancer Institute Giovanni Paolo II, Bari, Italy; ^10^ Radiology Unit, Bufalini Hospital, AUSL Romagna, Cesena, Italy; ^11^ Unit of Medical Oncology, Hospital of Faenza, AUSL Romagna, Faenza, Italy; ^12^ Department of General Surgery, Morgagni-Pierantoni Hospital, AUSL Romagna, Forlì, Italy; ^13^ Department of General Surgery, Infermi Hospital, AUSL Romagna, Rimini, Italy

**Keywords:** hepatocellular carcinoma, liver cancer, sorafenib, hypertension, predictive biomarker

## Abstract

Hypertension (HTN) is frequently associated with the use of angiogenesis inhibitors targeting the vascular endothelial growth factor pathway and appears to be a generalized effect of this class of agent. We investigated the phenomenon in 61 patients with advanced hepatocellular carcinoma (HCC) receiving sorafenib. Blood pressure and plasma electrolytes were measured on days 1 and 15 of the treatment. Patients with sorafenib-induced HTN had a better outcome than those without HTN (disease control rate: 63.4% vs. 17.2% (p=0.001); progression-free survival 6.0 months (95% CI 3.2-10.1) *vs.* 2.5 months (95% CI 1.9-2.6) (p<0.001) and overall survival 14.6 months (95% CI9.7-19.0) *vs*. 3.9 months (95% CI 3.1-8.7) (p=0.003). Sodium levels were generally higher on day 15 than at baseline (+2.38, p<0.0001) in the group of responders (+4.95, p <0.0001) compared to patients who progressed (PD) (+0.28, p=0.607). In contrast, potassium was lower on day 14 (−0.30, p=0.0008) in the responder group (−0.58, p=0.003) than in those with progressive disease (−0.06, p=0.500). The early onset of hypertension is associated with improved clinical outcome in HCC patients treated with sorafenib. Our data are suggestive of an activation of the renin-angiotensin system in patients with advanced disease who developed HTN during sorafenib treatment.

## INTRODUCTION

Hepatocellular carcinoma (HCC), the most common primary liver cancer, is increasing in incidence. It currently represents the fifth most common malignancy worldwide and the third leading cause of cancer-related death [1]. The introduction of sorafenib has positively changed the clinical landscape of the disease despite its limited efficacy and moderate toxicity in a substantial percentage of patients [2-7].

Hypertension (HTN) is frequently associated with the use of angiogenesis inhibitors targeting the vascular endothelial growth factor (VEGF) pathway and appears to be a generalized effect of this class of agent [8-14]. Physiologically, HTN develops when VEGF stimulates the production of nitric oxide and prostacyclins in vascular endothelial cells [15-17], inhibiting vasodilatory mechanisms, increasing peripheral vascular resistance and leading to higher blood pressure (BP). Moreover, vasoconstriction determines a decrease in the glomerular filtration rate and an increase in sodium and water retention by the kidneys, as occurs in pre-eclampsia which has been linked to placental-derived soluble antiangiogenic factors including VEGF [18,19].

The inhibition of VEGFR-2 by sorafenib leads to a decrease in phosphoinositide 3-kinase (PI3K), Akt, endothelium-derived nitric oxide synthase (eNOS) expression and the production of the potent vasodilator nitric oxide [20-22].

Based on these observations, we performed a retrospective analysis to evaluate whether the development of HTN and changes in serum electrolytes in patients with metastatic HCC receiving sorafenib are associated with the antitumor efficacy of the drug.

## RESULTS

### Patient characteristics

From 1 July 2011 to 25 March 2015, 61 consecutive patients with Child-Pugh A HCC receiving sorafenib were available for our analysis. Fifty-one (84.3%) were males and 10 (15.7%) females, with a median age at diagnosis of 72 years (range 28-87). Median follow-up was 34 months (range 1-45). Seven patients had BCLC-B and 54 had BCLC-C. Twenty (32%) patients had previously undergone transarterial chemo-embolisation (TACE). The most common liver disease etiologies were hepatitis C (50.8%), alcoholic liver disease (10.2%), fatty liver disease (15%) and hepatitis B (15.2%) (Table 1). The dose of sorafenib was reduced in 32 (52%) patients, 9 of whom had grade 3 HTN, 6 grade 3 HTN and grade 2 skin toxicity, 7 grade 2-3 skin toxicity, 5 grade 2-3 asthenia, and 5 grade 2-3 diarrhea. Median PFS was 2.8 months (95% CI 2.5-3.7) and median OS was 8.7 months (95% CI 5.7-13.9).

**Table 1:** Patient characteristics (n=61)

Variable	No. (%)
**Median age**, years (range)	72 (28-87)
**Gender**	
Male	51 (83.6)
Female	10 (16.4)
**ECOG PS**	
0	37 (60.7)
1	24 (39.3)
**Etiology**	
Hepatitis C	30 (50.8)
Hepatitis B	9 (15.2)
Alcoholic liver disease	6 (10.2)
Metabolic syndrome	10 (16.9)
Other	4 (6.8)
Missing	2
**BCLC Staging**	
B	7 (11.5)
C	54 (88.5)
**Vascular invasion**	
No	14 (40.0)
Si	21 (60.0)
Missing	26
**Pretreatment blood pressure**	
Mean systolic value (SD)	118.44 (11.82)
Mean diastolic value (SD)	76.15 (9.24)

BCLC, Barcelona Clinic Liver Cancer; SD, standard deviation

### Hypertension and clinical outcome

**Figure 1:**
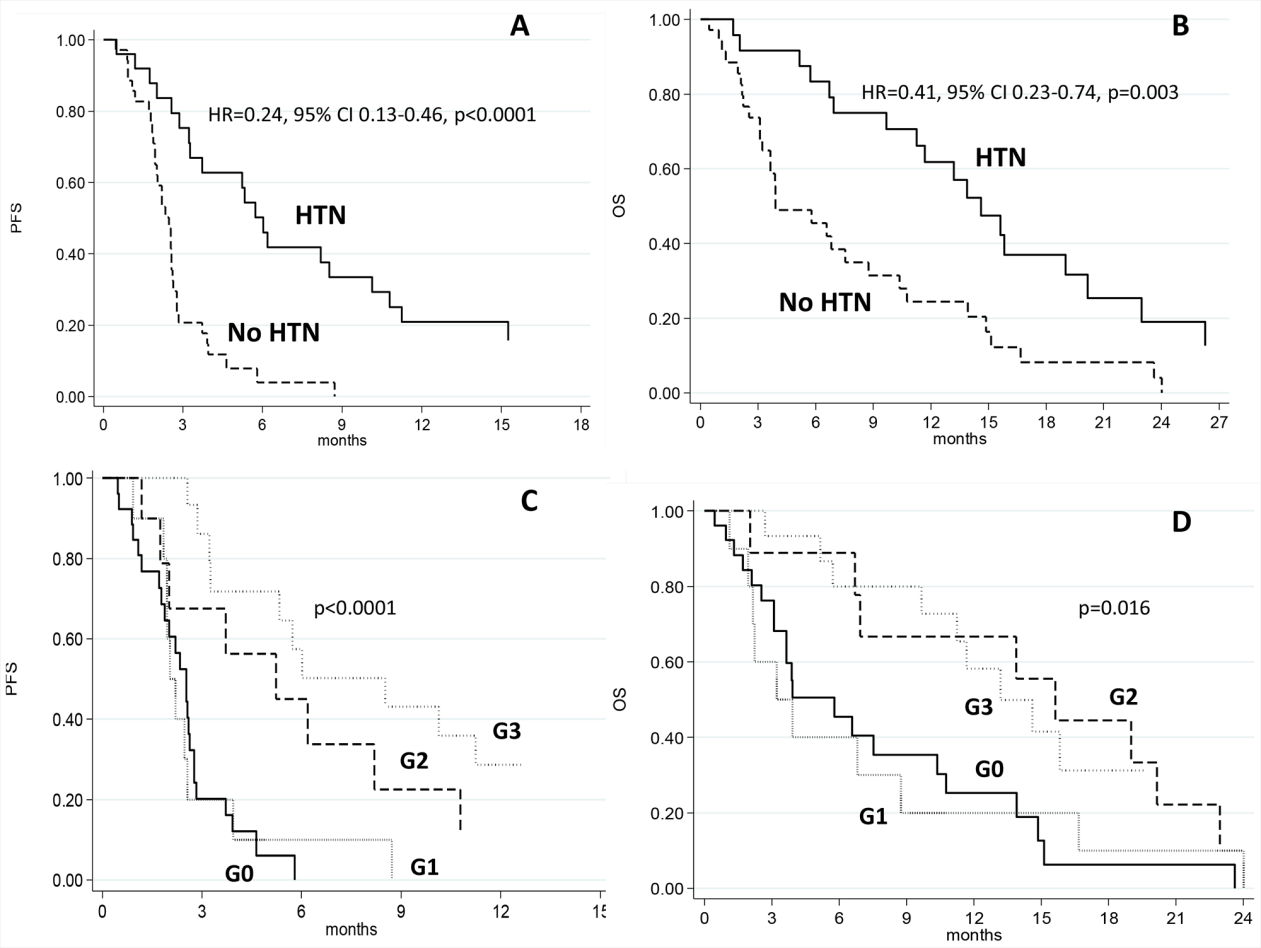
Progression-free and overall survival in patients with or without hypertension (HTN) A, C. and according to the grade of hypertension B, D

Patients who developed HTN after 15 days of treatment had a median PFS of 6 months (95% CI 3.2-10.1) compared to 2.5 months (95% CI 1.9-2.6) for those who did not (HR=0.24, 95% CI 0.13-0.46, p<0.0001) (Figure 1A). HTN patients had a median OS of 14.6 months (95% CI 9.7-19.0) compared to 3.9 months (95% CI 3.1-8.7) for those in the non HTN group (HR=0.41, 95% CI 0.23-0.74, p=0.003) (Figure 1B). DCR in HTN patients was 63.4% compared to 17.2% in those without HTN (p=0.001) (Table 2).

**Table 2:** Best objective response to sorafenib

	All patients (n=61) No. (%)	No HTN (n=35) No. (%)	HTN (n=26) No. (%)	p
**CR**	1 (2.0)	0	1 (4.5)	
**PR**	3 (5.9)	0	3 (13.6)	
**SD**	15 (29.4)	5 (17.2)	10 (45.5)	
**PD**	32 (62.7)	24 (82.8)	8 (36.4)	
**Missing/NE**	10	6	4	
**DCR** (CR+PR+SD)	19 (37.3)	5 (17.2)	14 (63.4)	0.001

HTN, hypertension; CR, complete response; PR, partial response; SD, stable disease; PD, progressive disease; NE, not evaluable; DCR, disease control rate

**Figure 2:**
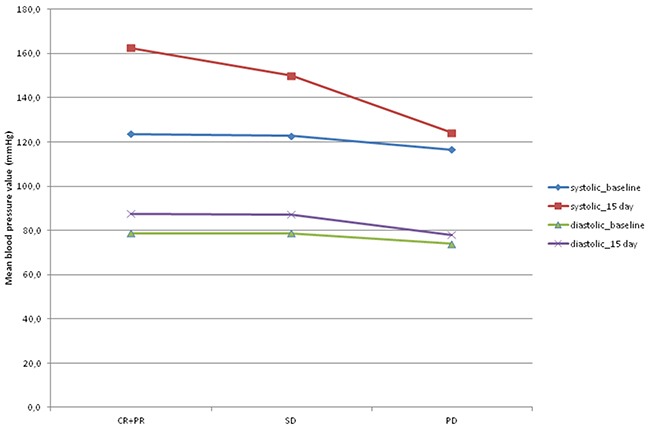
Mean values of systolic and diastolic blood pressure at baseline and on day 15 of the first cycle of sorafenib according to objective response CR+ PR: complete response plus partial response; SD: stationary disease; PD: progressive disease.

Figure 2 shows the mean values of SBP and DBP at baseline and on day 15 of the first cycle of sorafenib with respect to objective response. SBP increased significantly from baseline to day 15 of the first cycle of sorafenib, with a mean increase of 31 mm Hg in patients showing an objective response (p=0.003), 22.2 mm Hg in those with SD (p=0.004), and 6.6 mm Hg in PD patients (p=0.022).

DBP did not change significantly from baseline to day 15 of the first cycle of sorafenib in any group, regardless of objective response. Median PFS was 2.1 months (95% CI 0.9-2.6) in patients with grade 1 HTN, 5.2 months (95% CI 1.2-10.8) in those with grade 2 HTN, and 8.5 months (95% CI 3.2-15.3) (p<0.0001) in the grade 3 HTN group (Table 3 and Figure 1C). Median OS was 3.6 months (95% CI 1.1-8.7) in grade 1 HTN patients, 15.6 months (95% CI 2.0-23.0) in those with grade 2 HTN, and 13.2 months (95% CI 5.7-34.2) (p<0.011) in the grade 3 HTN group (Table 3 and Figure 1D). Comorbidities that could potentially influence BP were similar in the two cohorts.

**Table 3:** Progression-free and overall survival evaluated according to the hypertension toxicity (grade)

Grade	No. patients	No. events	Median PFS (months) (95% CI)	p	HR (95% CI)	p
**0**	26	24	2.5 (1.8-2.8)		1.00	
**1**	10	10	2.1 (0.9-2.6)		0.88 (0.41-1.87)	
**2**	10	9	5.2 (1.2-10.8)		0.30 (0.13-0.71)	
**3**	15	12	8.5 (3.2-15.3)	<0.0001	0.16 (0.07-0.37)	<0.0001

Grade	No. patients	No. events	Median OS (months) (95% CI)	p	HR (95% CI)	p
**0**	26	21	5.8 (3.1-10.4)		1.00	
**1**	10	10	3.6 (1.1-8.7)		0.96 (0.45-2.08)	
**2**	10	9	15.6 (2.0-23.0)		0.45 (0.20-1.01)	
**3**	15	11	13.2 (5.7-34.2)	0.011	0.32 (0.14-0.71)	0.016

HZ, hazard ratio

After adjusting for clinical covariates (age, gender, etiology, BCLC stage, ECOG performance status, toxicity, treatment duration and previous TACE), HTN remained an independent prognostic factor for both PFS (HR=0.21, 95% CI 0.09-0.48, p= 0.0002) and OS (HR=0.41, 95% CI 0.17-0.96, p=0.041).

### Hypertension and electrolyte changes

Comparing electrolyte values at baseline and on day 15 (Table 4), we observed a reduction in potassium levels (mean 5.7%) and an increase in sodium levels (average 1.6%) that were more marked in DCR patients (defined as responders) than in those with PD. Considering the trend of average electrolyte values of the 2 blood tests, the t-test for paired data showed that the absolute values of sodium on day 15 were generally higher than those at baseline (+2.38, p<0.0001) in DCR patients (+4.95, p<0.0001) compared to the PD group (+0.28, p=0.607). Potassium showed the same trend, with generally lower average values on day 15 than at baseline (−0.30, p=0.0008) in the DCR group (−0.58, p=0.003) than in PD patients (−0.06, p=0.500).

**Table 4:** Variation in sodium and potassium levels as a function of best objective response to sorafenib

	No. patients	Percentage variation after 15 days	
		Median value (IQR)	Median value (SD)
**Sodium**			
Overall	61	0 (−3 to 12.3)	1.6 (2.8)
DCR	19	3 (0 to 8.2)	3.3 (2.5)
PD	32	0 (−3 to 5.1)	0.25 (1.8)
p (between DCR and PD)		<0.0001^1^	
**Potassium**			
Overall	61	−2 (−41 to 38.13)	−5.7 (13.4)
DCR	19	−16 (−37 to 38.20)	−11.1 (16.0)
PD	32	0.50 (−41 to 14. 7)	−1.06 (10.2)
p (between DCR and PD)		0.0007^1^	

IQR, interquartile range; DCR, disease control rate; PD, progressive disease; SD, standard deviation

1 Wilcoxon median test

## DISCUSSION

**Figure 3:**
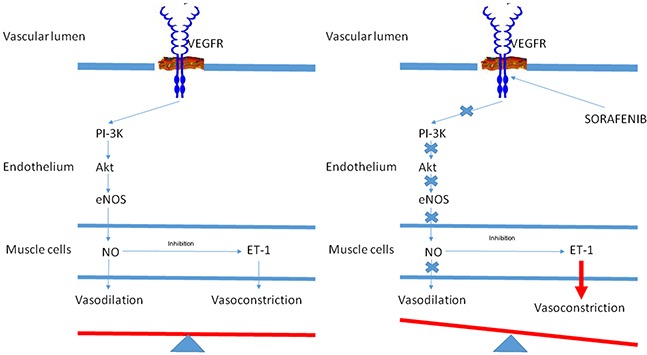
Hypothetic effect of vascular endothelial growth factor receptor (VEGFR) on blood vessel tone in patients A without sorafenib-induced hypertension and B. with sorafenib-induced hypertension.

In this retrospective study, a correlation emerged between the development of HTN during treatment with sorafenib and survival of advanced HCC patients. Patients with HTN also had a better DCR than those without HTN. Furthermore, we noticed that the higher the BP, especially systolic, the better the response to treatment with sorafenib. Figure 3 shows the hypothetic effect of VEGFR on blood vessel tone in patients (A) without sorafenib-induced HTN and (B) with sorafenib-induced HTN. The activation of VEGFR-2 via phosphoinositide 3-kinase (PI3K) and its downstream serine protein kinase Akt stimulates endothelium-derived nitric oxide synthase (eNOS), produce nitric oxide (NO), a potent vasodilatator. NO also exerts an inhibitory effect on the vasoconstrictor endothelin-1 (ET-1) (panel A). In patients treated with sorafenib, the inhibition of VEGFR-2 is thought to reduce the bioavailability of NO, resulting in vasoconstriction and consequent HTN [24-27].

Although a correlation between HTN and response to sorafenib in HCC patients has already been reported in some studies [28, 29], it was established on the basis of grade of toxicity alone by assessing HTN one or 2 months after the start sorafenib. Conversely, we evaluated SBP and DBP separately after only 15 days of treatment and not only in terms of grade of toxicity.

**Figure 4:**
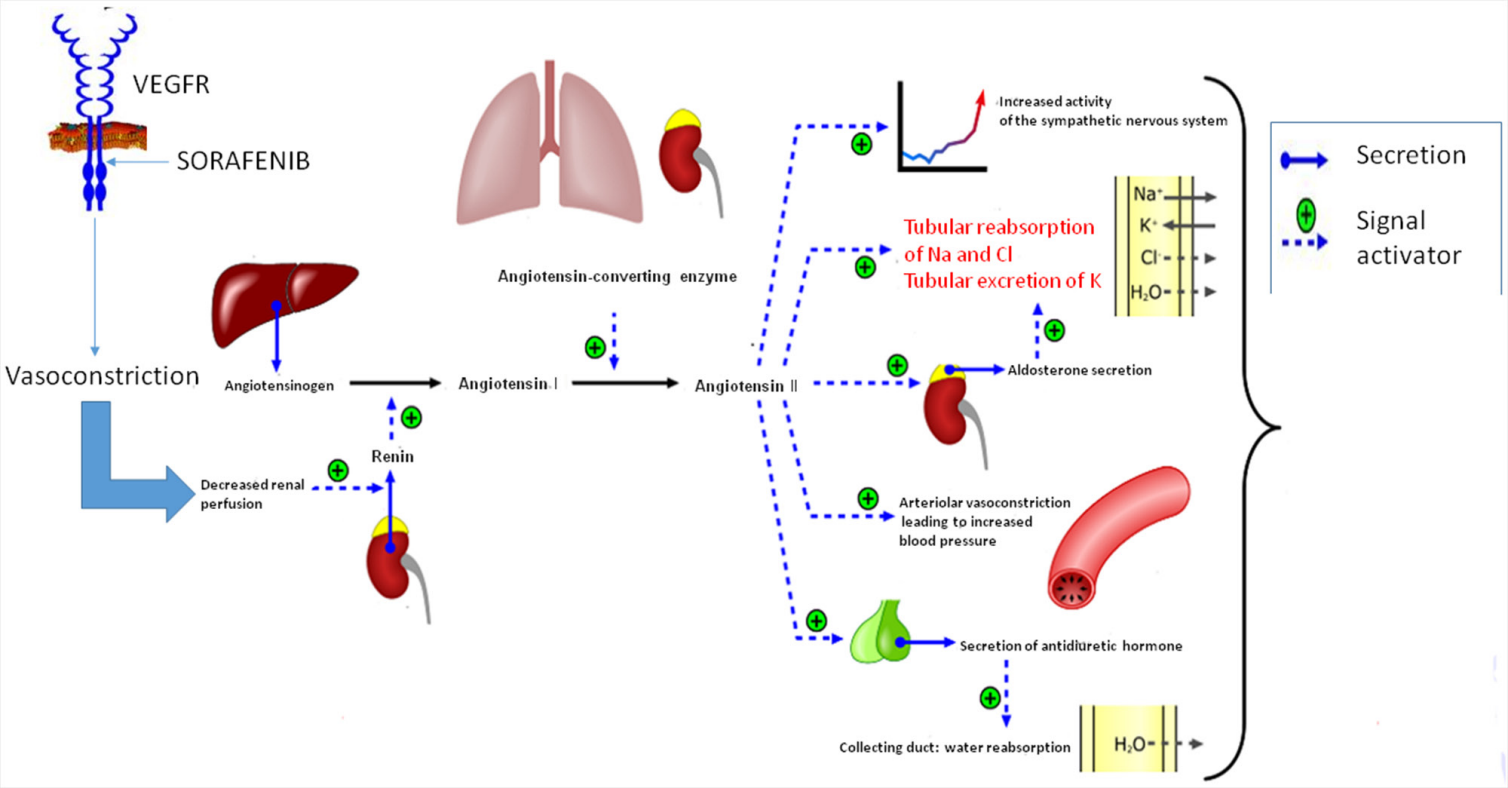
Hypothetic activation of the renin-angiotensin system in patients who develop HTN during treatment with sorafenib

Our data revealed a correlation between the change in electrolyte values and sorafenib-related HTN. It can be hypothesized that sorafenib blocks the VEGF receptor, inducing vasoconstriction in the kidneys, activating the renin-angiotensin-aldosterone system and increasing the production of renin. Renin converts angiotensin I into angiotensin II, leading to water retention, hypernatremia and hypokalemia [30] (Figure 4). Our data suggest that HTN in HCC patients undergoing treatment with sorafenib could be treated with ACE inhibitors.

One strength of our study lies in the detailed information it provides on patient characteristics and follow up. However, the study also has some limitations. Despite being a retrospective evaluation, cases were selected consecutively to minimize bias. Secondly, as there was no control arm (patients not receiving sorafenib), a clear distinction between the prognostic and predictive role of HTN in terms of survival could not be made. Although multivariable analysis indicated that HTN was an independent variable, it cannot be ruled out that results were biased by the relatively small sample number.

In conclusion, sorafenib-related HTN is potentially predictive factor associated with significantly longer OS and PFS in patients with advanced HCC.

## PATIENTS AND METHODS

This retrospective cohort study included all the patients treated for HCC between 1^st^ July 2013 and 25^th^ March 2015 at our institute (IRST IRCCS) in Meldola, Italy. Seventy-eight patients with advanced or intermediate stage HCC that was refractory or no longer amenable to locoregional therapies (according to American Association for the Study of Liver Diseases guidelines) were eligible for our analysis. All patients received a standard sorafenib regimen (400 mg bid continuously), dose reductions being made if deemed clinically necessary. Follow-up consisted of CT/MRI scanning every 8 weeks or as clinically indicated. Tumor response was evaluated by modified Response Evaluation Criteria in Solid Tumors (mRECIST) [23]. Treatment with sorafenib was continued until disease progression, unacceptable toxicity or death. Sorafenib was suspended in patients experiencing grade 4 treatment-related HTN (according to Common Terminology Criteria for Adverse Events, CTCAE, v4.03), while a dose reduction was recommended for patients with grades 1 to 3 HTN when blood pressure was difficult to control.

Patients were monitored as follows: days 1, 15, 28 and 60 of cycle 1, and every 2 months thereafter. Disease progression was assessed using mRECIST. Electrolytes were measured on days 1 and 15 of treatment. HCC patients without cirrhosis were excluded from the study. BP was measured on days 1 and 15 of treatment. Patients who had uncontrolled blood pressure at baseline (systolic BP >140 mm Hg and diastolic BP >90 mm Hg) were excluded from the study. HTN was defined as either a maximum or mean systolic BP (SBP) of at least 140 mm Hg or diastolic BP (DBP) of at least 90 mm Hg. HTN grade was defined according to the Common Terminology Criteria for Adverse Events (CTCAE) of the National Cancer Institute, version 4.03 of 14^th^ June 2010.

This study was approved by the local Ethics Committee and informed consent was obtained from all patients.

### Efficacy and safety assessments

The primary objective of this study was to compare progression-free survival (PFS) between patients who developed HTN and those who showed no signs of HTN during the first 15 days of treatment with sorafenib. Our second objective was to evaluate changes in blood electrolytes (sodium and potassium) in patients with HTN compared to those without HTN during the same period. We also planned to compare overall survival (OS) and disease control rate (DCR) between the 2 groups.

### Statistical methods

Appropriate descriptive statistics were used for demographic and tumor characteristics. Mean, median, standard deviation and minimum and maximum values were reported for continuous variables, while count and proportion were reported for non-continuous variables. PFS was defined as the time from the start of sorafenib therapy until disease progression or death from any cause or last follow-up visit. OS was defined as the time-interval between the start of sorafenib therapy and death from any cause or last follow-up visit. PFS, OS and their two-sided 95% confidence intervals (95% CI) were estimated by the Kaplan-Meier method and curves were compared by the log-rank test (at a significance level of 5%). DCR was defined as the proportion of patients to achieve complete response (CR), partial response (PR), stable disease (SD) or progressive disease (PD) according to mRECIST.

Estimated hazard ratios (HRs) and their two-sided 95% CI were calculated using the Cox proportional-hazard model. After univariate analysis, a multivariable Cox regression model (including age, gender, etiology, BCLC stage, ECOG performance status, toxicity, treatment duration and previous TACE) was used to adjust for these potential confounding factors. We investigated the potential association between HTN/blood electrolyte levels and disease progression, in particular using nonparametric tests to examine the relationship between changes in raw data and disease status of the 2 sequential blood tests.

All p values were based on two-sided testing and statistical analyses were performed using SAS statistical software version 9.3 (SAS Inc., Cary, NC, USA).
